# Polyphenol oxidase genes in barley (*Hordeum vulgare* L.): functional activity with respect to black grain pigmentation

**DOI:** 10.3389/fpls.2023.1320770

**Published:** 2024-01-08

**Authors:** Anastasiia Y. Glagoleva, Tat’jana V. Kukoeva, Elena K. Khlestkina, Olesya Y. Shoeva

**Affiliations:** ^1^ Institute of Cytology and Genetics (ICG), Siberian Branch of Russian Academy of Sciences (SB RAS), Novosibirsk, Russia; ^2^ N.I. Vavilov All-Russian Research Institute of Plant Genetic Resources (VIR), Saint Petersburg, Russia

**Keywords:** *Black lemma and pericarp 1*, enzymatic browning, epistasis, genetic segregation, melanin

## Abstract

Polyphenol oxidase (PPO) is an oxidoreductase. In damaged plant tissues, it catalyzes enzymatic browning by oxidizing *o*-diphenols to highly reactive *o*-quinones, which polymerize producing heterogeneous dark polymer melanin. In intact tissues, functions of PPO are not well understood. The aim of the study was to investigate the barley PPO gene family and to reveal the possible involvement of *Ppo* genes in melanization of barley grain, which is controlled by the *Blp1* gene. Based on known barley *Ppo* genes on chromosome 2H (*Ppo1* and *Ppo2*), two additional genes—*Ppo3* and *Ppo4*—were found on chromosomes 3H and 4H, respectively. These genes have one and two exons, respectively, contain a conserved tyrosinase domain and are thought to be functional. Comparative transcriptional analyzes of the genes in samples of developing grains (combined hulls and pericarp tissues) were conducted in two barley lines differing by melanin pigmentation. The genes were found to be transcribed with increasing intensity (while grains mature) independently from the grain color, except for *Ppo2*, which is transcribed only in black-grained line i:Bw*Blp1* accumulating melanin in grains. Analysis of this gene’s expression in detached hulls and pericarps showed its elevated transcription in both tissues in comparison with yellow ones, while it was significantly higher in hulls than in pericarp. Segregation analysis in two F_2_ populations obtained based on barley genotypes carrying dominant *Blp1* and recessive *ppo1* (I) and dominant *Blp1* and recessive *ppo1* and *ppo2* (II) was carried out. In population I, only two phenotypic classes corresponding to parental black and white ones were observed; the segregation ratio was 3 black to 1 white, corresponding to monogenic. In population II, aside from descendants with black and white grains, hybrids with a gray phenotype — light hulls and dark pericarp — were observed; the segregation ratio was 9 black to 3 gray to 4 white, corresponding to the epistatic interaction of two genes. Most hybrids with the gray phenotype carry dominant *Blp1* and a homozygous recessive allele of *Ppo2*. Based on transcription and segregation assays one may conclude involvement of *Ppo2* but not *Ppo1* in melanin formation in barley hulls.

## Introduction

Polyphenol oxidases (PPO) are oxidoreductase enzymes widespread among plant species. Enzymes of this class share a tyrosinase domain that contains two conserved copper-binding motifs: CuA and CuB (Marusek et al., 2006). PPOs can exert two types of activity: monophenolase (E.C. 1.14.18.1), which catalyzes the *o*-hydroxylation of monophenols forming *o*-diphenols, and diphenolase (E.C. 1.10.3.), which oxidizes *o*-diphenols to *o*-quinones. The highly reactive *o*-quinones are then polymerized nonenzymatically giving rise to dark-colored compounds: melanins (Nicolas et al., 1994). PPOs located in plastids are thought to be spatially separated from their phenolic substrates, which are found in vacuoles of plant cells (Vaughn and Duke, 1984). Therefore, oxidation of phenolic substrates to melanins usually occurs after a mechanical impact damaging cell compartments. This process is called the enzymatic browning reaction and considered to be an important part of the plant immune response to stress (Zhang, 2023). However, the undesirable darkening of plants caused by the enzymatic browning reaction during the postharvest process has substantial negative effects on commercial value of agricultural products (Taranto et al., 2017; Moon et al., 2020). One way to avoid this is to breed varieties with inactive PPO; this approach requires knowledge about functions of *Ppo* genes in different plant tissues and about allelic diversity of these genes.

Besides enzymatic browning, additional functions in plant physiology are attributed to PPO and should be taken into consideration when *Ppo*-deficient varieties are bred. For example, it was shown that a knockout of the single gene encoding PPO results in the appearance of dark necrotic spots on leaves of walnut (*Juglans regia*), and this phenomenon was associated with disruption of tyrosine metabolism and accumulation of a toxic compound called tyramine in cells (Araji et al., 2014). On the other hand, a knockout of *Ppo* genes in potato (*Solanum tuberosum*) also has a significant effect on metabolism of phenolic compounds, thereby increasing accumulation of end products of the phenylpropanoid biosynthetic pathway (in the mutant lines compared to the wild type) and enhancing resistance to late blight (Llorente et al., 2011; Llorente et al., 2014). Given that PPO is localized to chloroplasts, possible participation of PPOs in photosynthesis and in protection of the photosynthetic apparatus from damage under abiotic stress conditions has been discussed (Boeckx et al., 2015; Boeckx et al., 2017). Additionally, PPO activity can be associated with genetically determined synthesis of constitutive melanin pigments in plant seed coats. Black pigmentation of plant seeds is a common trait, and for some plant species, melanin presence has been confirmed by chemical extraction and identification methods (Glagoleva et al., 2020). For example, melanin has been isolated from hulls of sunflower seeds (Nicolaus et al., 1964; Gracheva and Zheltobryukhov, 2016), watermelon seeds (Nicolaus et al., 1964), and chestnuts (Yao et al., 2012), sesame (Dossou et al., 2022) as well as from seeds of cereals like oat (Varga et al., 2016), barley (Shoeva et al., 2020), and wheat (Bazhenov et al., 2023).

Melanin is the least investigated plant pigment owing to its nonobvious functions and laborious extraction. Nevertheless, it is believed that the dark color of seeds provides some advantages to plants during adaptation to unfavorable environmental conditions (Glagoleva et al., 2020). For example, the barley landraces with black seeds are grown in the most arid regions of Syria, whereas white-grained landraces are adapted to milder growing conditions (Ceccarelli et al., 1987). Specimens with black grains have turned out to be more cold- and drought-tolerant than specimens with white grains (Ceccarelli et al., 1987; Weltzien, 1988). Besides abiotic stress, melanin is believed to participate in protection of plants from biotic stress. Varieties of oat with a dark spike color caused by melanin are less affected by *Fusarium* infection than are varieties without dark husk pigments (Loskutov et al., 2016). Barley recombinant inbred lines with black grains demonstrated lower incidence of *Fusarium* head blight and lower accumulation of the mycotoxin deoxynivalenol than did lines with yellow grains (Choo et al., 2015). Although melanin is a widespread pigment in the plant kingdom, there is little information on how it is synthesized. In some plant species, a possible relation between the activity of *Ppo* genes and the biosynthesis of melanin pigments in intact tissues has been reported. For example, in watermelon, the gene encoding PPO was identified as a candidate gene responsible for melanin synthesis (Li et al., 2020). Comparative transcriptome studies on peanut (Wan et al., 2016) and sesame (Wang et al., 2020) have revealed increased expression of genes encoding PPO in melanin-accumulating seeds. In addition, an involvement of PPO in melanin synthesis in persimmon peel was suggested (Qi et al., 2020).

Barley (*Hordeum vulgare* L.) is one of the most widely cultivated crops in the world. It is believed that the presence of melanin in the hulls of barley seeds is a common characteristic of barley populations (Glagoleva et al., 2022). The black color of the barley grain is under monogenic control by the *Blp1* (*Black lemma and pericarp 1*) locus on chromosome 1H (Costa et al., 2001; Long et al., 2019). Based on existing knowledge about the locus, it can be argued that the gene determining melanin synthesis in the barley grain does not encode PPO (Jia et al., 2017; Long et al., 2019), but in a comparative RNA-seq analysis of white- and black-grained near-isogenic lines (NILs), the *Blp1* locus was shown to affect the expression of the *Ppo* genes that have been proposed as components of a gene network controlling melanin formation in the barley grain (Glagoleva et al., 2017). Two genes encoding PPO have already been found in the barley genome, which are referred to as *Ppo1* and *Ppo2* and are localized to the long arm of chromosome 2H (Taketa et al., 2010). Besides them, two additional *Ppo* genes are thought to exist in the barley genome because of the presence of two additional *Ppo* genes on chromosomes 3H and 4H in the related wild barley species *Hordeum chilense* (Rodríguez-Suárez and Atienza, 2014). However, functions of the *Ppo* genes with respect to melanin formation in the barley grain have not been studied. Thus, the aim of the study was to investigate the barley polyphenol oxidase gene family and to reveal the possible involvement of *Ppo* genes in melanin synthesis in the barley grain.

## Materials and methods

### Plant material

NIL i:Bw*Blp1* (also known as BW062; accession number in NordGen collection: NGB20470; www.nordgen.org) carrying the *Blp1.b* allele of the *Blp1* (*Black lemma and pericarp 1*) locus mapped to chromosome 1HL (Druka et al., 2011) and characterized by melanin accumulation in the husk and grain pericarp was used for the study. The NIL was developed in the genetic background of spring cultivar Bowman (“Bowman From Fargo,” NGB22812), which features the absence of melanin pigmentation in the grain. Two NILs with naked caryopsis that were designated as i:Bw*nud1* and i:Bw*Blp1nud1* were also employed in the study, which are characterized by unpigmented and melanin-containing grains, respectively. These lines were developed based on Intense blue aleurone (NGB20651, characterized by naked caryopsis and the absence of melanin in the grain) and i:Bw*Blp1* lines. The scheme of obtaining of lines is presented in **Supplementary Figure 1**. Barley specimens carrying mutant alleles of genes *Ppo1* and *Ppo2* served as parental ones for segregation analysis. Variety 4970 (U004, Institute of Plant Science and Resources, Okayama University, Japan, BarleyDB, http://earth.nig.ac.jp/~dclust/cgi-bin/index.cgi?lang=en) has a recessive allele of the *Ppo1* gene, and variety Shogor 3 (C 97-3) (I677) carries both recessive *Ppo1* and *Ppo2* genes (Taketa et al., 2010). Plants were grown in a greenhouse at the Institute of ICG SB RAS (Novosibirsk, Russia) under a 12-h photoperiod in a temperature range of 20–25°C.

### Identification of *Ppo* genes and *in silico* sequence analysis

The BLAST algorithm run against databases NCBI (https://www.ncbi.nlm.nih.gov/) and Ensembl Plants (https://plants.ensembl.org/index.html) was carried out to identify sequences of *Ppo* genes in genomes of barley and other cereals with 80% homology as a selection criterion. Tyrosinase domain sequences of genes *Ppo1* (AB549330) and *Ppo2* (AB549331) from ref (Taketa et al., 2010) were used as a query. Multiple sequence alignment was performed in the MUSCLE software (Madeira et al., 2019). Gene structure was predicted using FGENESH+ (Solovyev, 2004). Prediction of genes’ functional domains was performed by means of the InterPro database (Mitchell et al., 2019). A phylogenetic tree was constructed in the MEGA X software (Kumar et al., 2018) by the neighbor-joining method with a bootstrap support of 1000. The IDT PrimerQuest tool (http://eu.idtdna.com/PrimerQuest/Home/) was used to design PCR primers. The full list of PCR primers utilized in this work can be found in **Supplementary Table 1**.

### Total-RNA extraction and gene expression analysis by quantitative reverse-transcription PCR (qRT-PCR)

Total RNA from i:Bw*Blp1* and cv. Bowman was isolated at three stages of spike development: from developing spikelets at the booting stage (46 days after sowing, BBCH code 49), from combined tissues of the grain pericarp and husk at the early dough stage (67 days after sowing, BBCH code 77), and at the soft-dough stage (71 days after sowing, BBCH code 83). Furthermore, total RNA from the hulls and pericarp (separated from each other by peeling with tweezers) of lines i:Bw*Blp1*, i:Bw*nud1*, i:Bw*Blp1nud1*, and cv. Bowman was isolated at the early dough stage (67 days after sowing, BBCH code 77). RNA was extracted from the samples as three biological replicates, each obtained by pooling material from three plants. The RNA isolation was performed using the RNeasy Plant Mini Kit (QIAGEN, Hilden, Germany) including DNase I treatment (QIAGEN RNase-Free DNase Set). RNA concentration in the final solution was determined on a NanoDrop™ 2000 spectrophotometer (Thermo Fisher Scientific Inc., Waltham, MA, USA). cDNA was synthesized from 1 μg of total RNA by reverse transcription with the (dT)_15_ primer and RevertAid™ Kit (Thermo Fisher Scientific Inc.). qRT-PCR was conducted by means of the SYNTOL SYBR Green I Kit (Synthol, Moscow, Russia) according to the manufacturer’s protocol. The amplification was performed using a QuantStudio 5 RealTime PCR System (Thermo Fisher Scientific Inc.). Each reaction was carried out in three technical replicates. Relative expression levels were calculated by the standard-curve method and normalized to *Actin* gene expression (von Zitzewitz et al., 2005). The Mann–Whitney *U* test was performed to evaluate the significance of differences in expression levels between samples.

### PPO activity assay

The total PPO activity in mature seeds was evaluated with L-tyrosine according to a method described by (Taranto et al., 2012) with modifications. Seeds of cv. Bowman and i:Bw*Blp1* line were ground and incubated in a 0.01 M L-tyrosine solution (1 g per 4 mL of the solution) at 37°C for 19 h; after centrifugation at 12000 rpm, absorbance of the collected supernatant was measured at 405 nm on a UV/visible spectrophotometer (Bio-Rad, USA). The reported data are presented as the average of three biological replicates per sample. The Mann–Whitney *U* test was performed to evaluate the significance of differences between samples.

### Segregation analysis

Specimens of barley 4970 and Shogor 3 were crossed with NIL i:Bw*Blp1* to obtain two F_2_ populations: 4970 × i:Bw*Blp1* (124 plants) and Shogor 3 × i:Bw*Blp1* (202 plants). The color of grains was determined visually after maturity, and the mode of inheritance of the trait was evaluated by the Chi-square test. DNA was extracted from leaves of each F_2_ plant according to the procedure described by (Plaschke et al., 1995) and was genotyped by PCR with primers that allow to distinguish alleles of genes *Ppo1*, *Ppo2* (Taketa et al., 2010), and *Blp1* followed by electrophoresis of amplicons in a 3% agarose gel (**Supplementary Table 1**).

## Results

### Identification of new members of the polyphenol oxidase gene family in barley

Based on tyrosinase domain sequences of *Ppo1* (AB549330) and *Ppo2* (AB549331), two additional *Ppo* genes were identified in the barley genome via a homology search by the BLAST algorithm: HORVU.MOREX.r3.3HG0294590, localized to the distal region of the long arm of chromosome 3H, and HORVU.MOREX.r3.4HG0418160, situated in the distal region of the long arm of chromosome 4H. These genes were designated as *Ppo3* and *Ppo4*, respectively.

**Figure 1:**
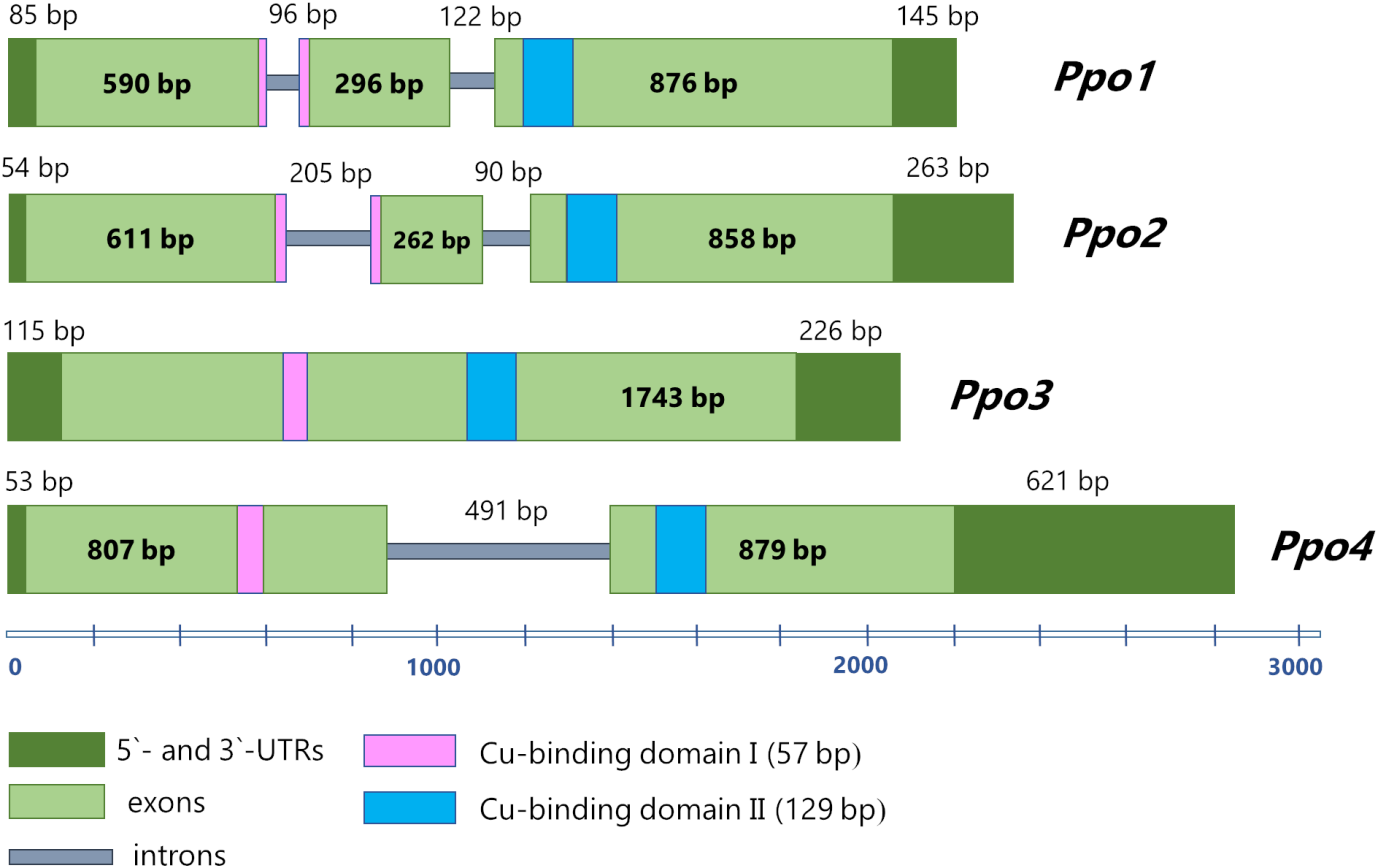
The structure of *Ppo* genes in barley. 3` and 5`-UTRs are designated by dark green, exons are light green, introns are grey. The Cu-binding domains I and II are highlighted by pink and blue, respectively.

The length of nucleotide sequence of the *Ppo3* gene amounted to 2084 bp, including a 1743 bp protein-coding region and 5′ and 3′ untranslated regions (115 and 226 bp, respectively). No introns were found in the *Ppo3* gene (**Figure 1**). The length of nucleotide sequence of the *Ppo4* gene is 2851 bp, including 5′ and 3′ untranslated regions (53 and 621 bp, respectively). The protein-coding part of the *Ppo4* gene consists of one intron (491 bp) and two exons (807 and 879 bp) (**Figure 1**). A comparison of structural organization of the barley polyphenol oxidase family genes indicated that genes *Ppo3* and *Ppo4* differ in their exon-intron structure from previously described genes *Ppo1* and *Ppo2*, which contain three exons and two introns each (**Figure 1**). Despite the differences in the structure of the identified *Ppo* genes, as well as their high variation within the family, all four genes have a functional tyrosinase domain containing two conserved Cu-binding motifs (**Supplementary Figure 2**).

### Phylogenetic analysis of *Ppo* genes in barley

**Figure 2:**
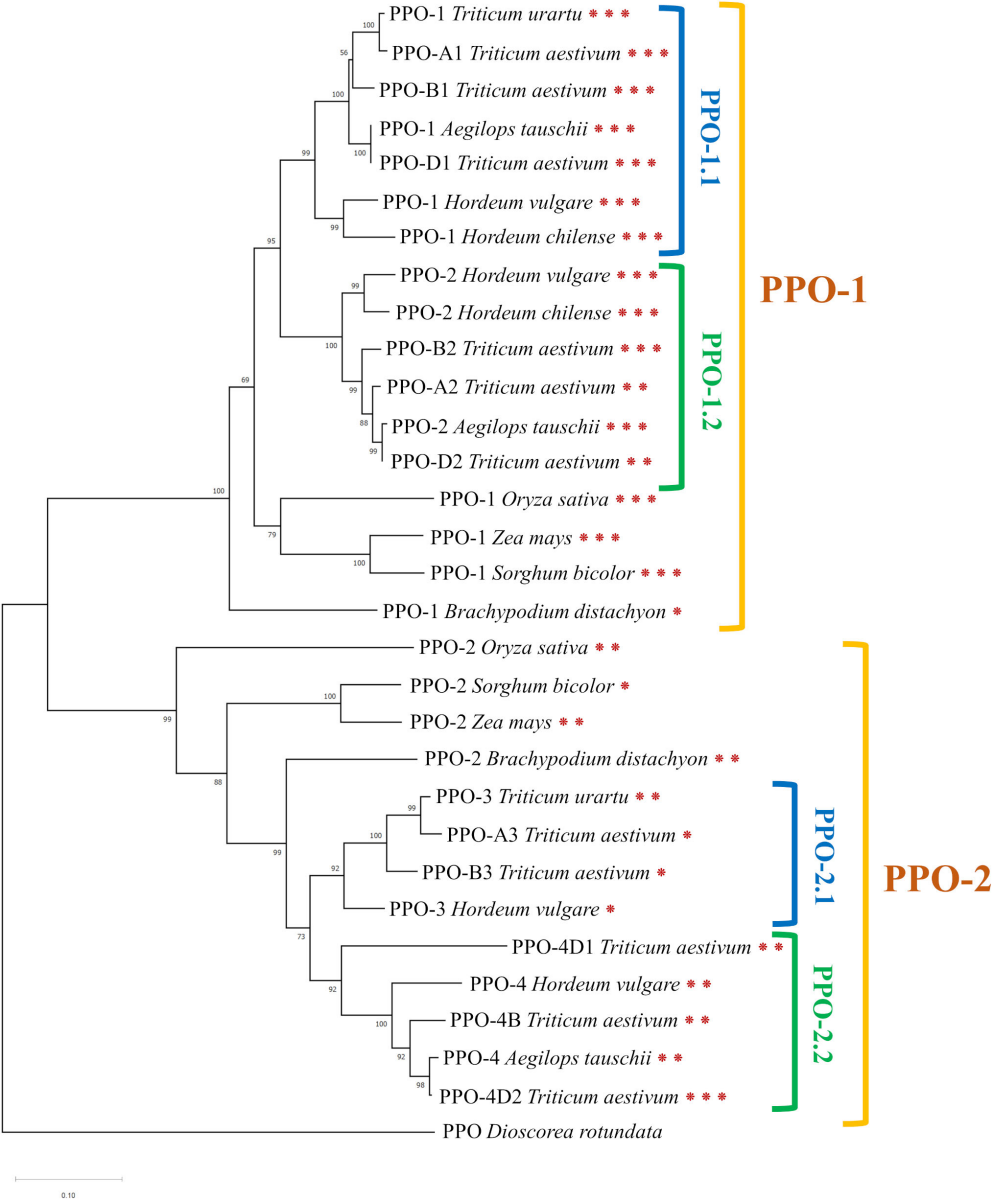
The phylogenetic tree constructed based on amino acid sequences of the tyrosinase domain of PPO (neighbor-joining method with a bootstrap support of 1000). Asterisks indicate the number of exons.

Tyrosinase domain sequences of *Ppo* genes from the following species were identified and used to construct a phylogenetic tree: *Triticum aestivum* (11 genes), *Triticum urartu* (two genes), *Aegilops tauschii* (three genes), *Hordeum chilense* (two genes), *Oryza sativa* (two genes), *Zea mays* (two genes), *Brachypodium distachyon* (two genes), and *Sorghum bicolor* (two genes). The PPO sequence of guinea yam (*Dioscorea rotundata*) served as an outgroup. The phylogenetic tree constructed from these sequences is displayed in **Figure 2**. The complete list of gene sequences with identification numbers from the Ensemble Plants database is provided in **Supplementary Table 2**.

*Ppo* genes of the cereal species under study are divided into two main clusters, between which two subclusters can be distinguished (**Figure 2**). The first cluster, designated as PPO-1, includes the *Ppo1* and *Ppo2* genes of barley species (*H. vulgare* and *H. chilense*) and highly homologous sequences of *Triticeae* species: *T. aestivum*, *A. tauschii*, and *T. urartu*. Besides, the PPO-1 cluster contains *Ppo* genes from rice (*O. sativa*), maize (*Z. mays*), *Brachypodium* (*B. distachyon*), and sorghum (*S. bicolor*). Within this cluster, the *Ppo* sequences of *Triticeae* species are divided into two subclusters, PPO-1.1 and PPO-1.2, represented by two groups of duplicated genes on chromosome 2.

In the second cluster, referred to as PPO-2, two subclusters can be distinguished too: PPO-2.1 and PPO-2.2 (**Figure 2**). The PPO-2.1 cluster includes the barley *Ppo3* gene and *Ppo* genes of *Triticeae* species located in the third group of chromosomes: PPO-3 of *T. urartu*, PPO-A3 of *T. aestivum*, and PPO-B3 of *T. aestivum*. The PPO-2.2 cluster includes the *Ppo4* gene of barley and homologous genes of *T. aestivum* and *A. tauschii* on chromosome 4. Just as the PPO-1 cluster, the PPO-2 cluster contains such genes of rice, maize, *Brachypodium*, and sorghum.

It was noted that genes in clusters PPO-1 and PPO-2 have different exon-intron structures (**Figure 2**). Most of the genes in the PPO-1 group have three exons, except for PPO-A2 and PPO-D2 genes of *T. aestivum*, which have two exons, and the PPO-1 gene of *B. distachyon*, whose sequence contains one exon. Genes in the PPO-2 group are generally characterized by the presence of two exons; however, *Ppo-2* of *S. bicolor* and genes from the PPO-2.1 subgroup have only one exon, with the exception of the PPO-3 gene of *T. urartu*. In the PPO-2.2 subgroup, the PPO-4D2 gene of *T. aestivum* has three exons.

These data suggested that the *Ppo3* and *Ppo4* genes identified in the barley genome are not unique and are represented by orthologous genes in other cereals.

### Expression analysis of *Ppo* genes and *PPO* activity assay

**Figure 3:**
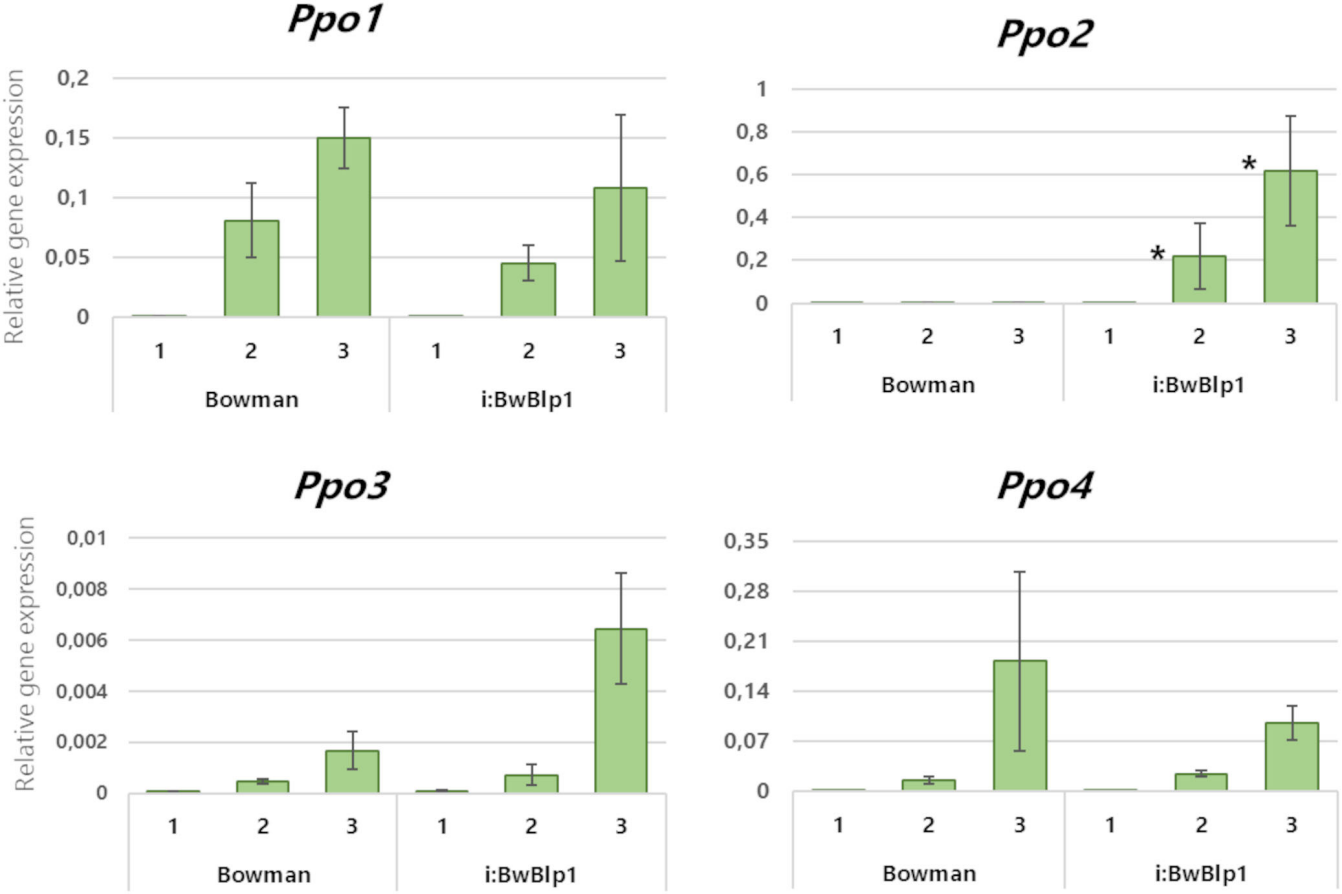
Relative expression levels of *Ppo* genes in combined tissues of the pericarp and lemma from hulled barley lines in the course of spike maturation. 1: Booting stage, 2: early dough stage, 3: soft-dough stage. *A difference is statistically significant (*U* test, *p* < 0.05) between i:Bw*Blp1* and cv. Bowman at the same developmental stage.

Comparative expression analysis of *Ppo* genes in the barley spike during maturation was performed next. The barley NIL i:Bw*Blp1*, which accumulates melanin pigments in the hulls and grain pericarp, and its melaninless parental cv. Bowman were used. The analysis was performed on tissues of the hulls and pericarp combined at each of three time points: the booting stage, early dough stage, and soft-dough stage, (**Figure 3**). None of the *Ppo* genes were found to be expressed at the booting stage regardless of the genotype, but expression increased as the plants matured. At the early dough and soft-dough stages, it was possible to detect differences in expression between *Ppo* genes depending on the presence of melanin. The transcriptional activity of genes *Ppo1* and *Ppo4* increases during spike development but does not differ significantly between i:Bw*Blp1* and cv. Bowman. The *Ppo2* gene is not transcriptionally active in Bowman at any of the stages examined, whereas elevated expression of this gene was registered in the melanin-accumulating line (**Figure 3**). Increased expression of the *Ppo3* gene in the i:Bw*Blp1* line in comparison with Bowman was observed, but the differences between the lines were not statistically significant.

**Figure 4:**
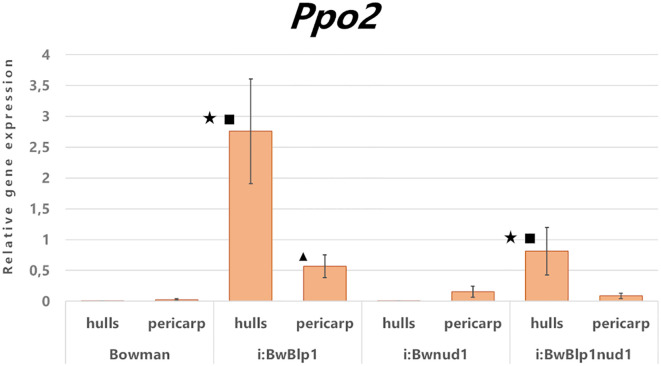
Relative expression levels of the *Ppo2* gene in detached tissues of the pericarp and hulls from hulled (Bowman, i:Bw*Blp1*) and naked (i:Bw*nud1*, i:Bw*Blp1nud1*) lines at the early dough stage. Star – a difference is statistically significant (*U* test, *p* < 0.05) between the hulls and pericarp within a line, square – a difference is statistically significant between lines with black and white hulls, triangle – a difference is statistically significant between lines with black and white pericarps.

Expression analysis of the *Ppo2* gene was performed on detached tissues of the hulls and pericarp at the early dough stage in hulled i:Bw*Blp1* and cv. Bowman and its naked derivatives: lines i:Bw*Blp1nud1* and i:Bw*nud1* (**Figure 4**). In hulled lines, elevated transcription of *Ppo2* was detected in both melanin-containing tissues—hulls and pericarp— in comparison with noncolored ones, while in naked lines, transcription of *Ppo2* was increased in melanin-containing hulls, but not in pericarp, in comparison with tissues of noncolored line. However, in both sets of lines (hulled and naked) *Ppo2* expression was significantly higher in melanin-containing hulls in comparison with the pericarp. It can be hypothesized that, of the four genes encoding PPO in barley, *Ppo2* may participate in melanin biosynthesis in barley grain.

To confirm the obtained comparative transcriptional data, the PPO activity assay was performed on mature grain of cv. Bowman and of i:Bw*Blp1* line. The observed activity of PPO in the i:Bw*Blp1* line was significantly higher in comparison with cv. Bowman: about two times higher (**Supplementary Figure 3**). Overall the observed increased expression of the *Ppo* genes in envelopes of black grains coincides with enhanced PPO activity in mature grains.

### Testing of *Ppo* involvement in melanin synthesis in the barley grain

**Figure 5:**
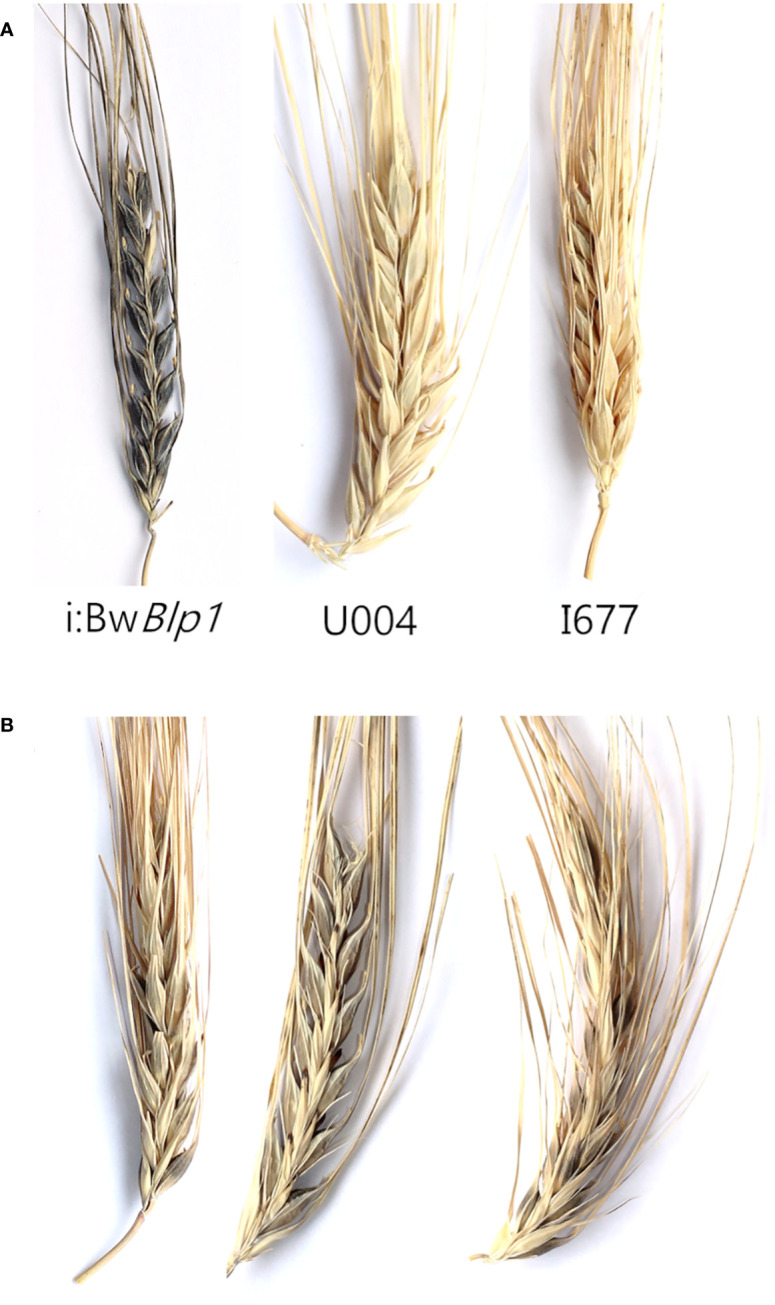
**(A)** Phenotypes of parental lines used in crosses, **(B)** the gray phenotype revealed among F_2_ plants in the Shogor 3 × i:Bw*Blp1* cross.

To evaluate the involvement of the *Ppo2* gene in melanin formation in the barley grain, two F_2_ populations were generated: 4970 × i:Bw*Blp1* (124 plants) and Shogor 3 × i:Bw*Blp1* (202 plants). Variety 4970 carries a recessive *Ppo1* gene resulting from a mutation leading to a stop codon in the first exon (Taketa et al., 2010). The Shogor 3 variety is characterized by both recessive *Ppo1* and *Ppo2* genes: it has a single-nucleotide insertion in the second exon of the *Ppo1* gene and a 8 bp insertion in the first exon of the *Ppo2* gene (Taketa et al., 2010). Both lines do not accumulate melanin in grain envelopes (**Figure 5A**).

**Figure 6:**
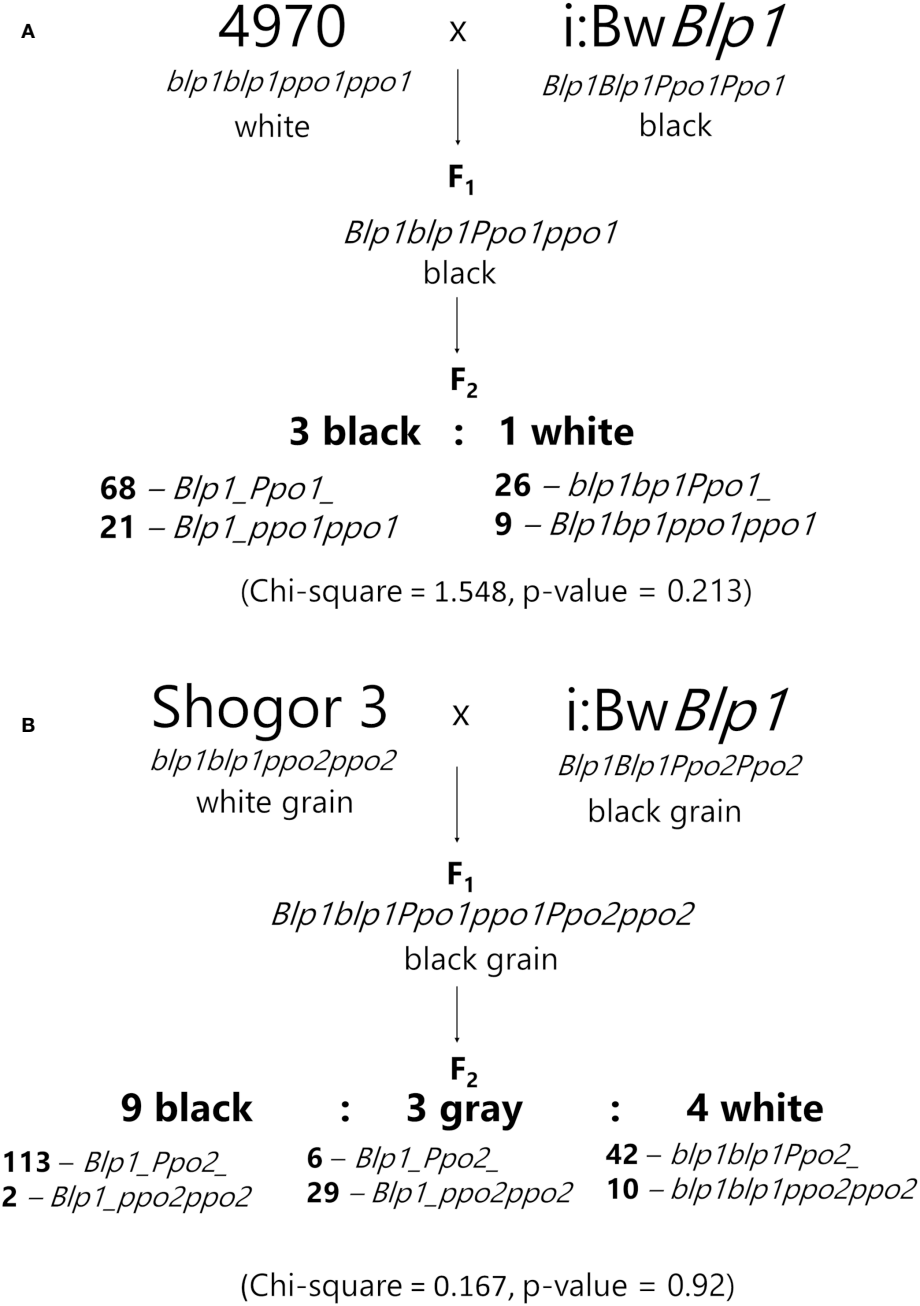
Segregation in F_2_ populations 4970 × i:Bw*Blp1* **(A)** and Shogor 3 × i:Bw*Blp1* **(B)**.

In F_1_, the black color of the hulls and grain pericarp was observed in all plants resulting from the crosses, just as in the parental i:Bw*Blp1* line. In the 4970 × i:Bw*Blp1* cross, F_2_ plants could be divided into two groups according to the grain color. The segregation ratio between melanin-pigmented and unpigmented plants was 3:1 (Chi-square = 1.548, p-value = 0.213). The F_2_ plants were genotyped with the primers for the *Ppo1* and *Blp1* genes, and the presence of melanin in the barley grain was found to depend only on the allelic state of the *Blp1* gene (not on alleles of the *Ppo1* gene; **Figure 6A**).

In the F_2_ population resulting from the Shogor 3 × i:Bw*Blp1* cross, three phenotypic classes were identified: the black color of the grain: corresponding to the i:Bw*Blp1* phenotype, gray: with a lighter gray color of the grain, and white: melanin was not observed in the grain, corresponding to the phenotype of Shogor 3 (**Figure 5B**). The black, gray, and white phenotypic classes were present in a ratio of 9:3:4 (Chi-square = 0.167, p-value = 0.92), which may indicate an epistatic interaction between genes *Ppo2* and *Blp1*. It turned out that 29 of the 35 plants with the gray phenotype had the *Ppo2* gene in the recessive homozygous state and a dominant allele of the *Blp1* gene in a homozygous or heterozygous state (**Figure 6B**, **Supplementary Figure 4**). All plants with melanin in the grain carried a dominant *Blp1* allele, and none of the plants characterized by the simultaneous presence of the dominant *Blp1* gene allele and the recessive *Ppo2* gene allele showed complete absence of melanin. Visual examination of hybrids carrying dominant *Blp1* and recessive *Ppo2* revealed that they had noncolored hulls and awns and dark pigments in underlying tissues. From these data, we can theorize that *Ppo2* is responsible for tissue-specific melanin accumulation in hulls.

## Discussion

PPO are well-researched plant enzymes. They carry out a protective enzymatic browning reaction that proceeds when the integrity of plant cell compartments is disrupted (Vaughn and Duke, 1984). PPO-induced browning of produce is undesirable, and therefore ways to prevent it are being actively sought, including the development of plant varieties with inactive PPO (Taranto et al., 2012; Hamdan et al., 2022). In barley, the strategies to avoid discoloration of barley-based food products include selecting genotypes with low discoloration such as proanthocyanidin-free genotypes, lowering the total polyphenol content or PPO activity through abrasion, heat treatment, exclusion of oxygen, and the use of enzyme inhibitors (Quinde-Axtell et al., 2006); breeding of PPO-deficient barley varieties is also possible because alleles of *Ppo* genes encoding inactive enzymes have been revealed in barley collections (Taketa et al., 2010).

Two *Ppo* genes mapped to the long arm of chromosome 2H have been found previously in the barley genome (Taketa et al., 2010). Here, we discovered two additional *Ppo* genes: *Ppo3* and *Ppo4*. They were shown to encode intact functional domains and to be expressed in the barley spike. During our phylogenetic analysis, orthologs of these genes were identified in other cereal species. It is known that the size of gene families encoding PPO can vary widely among plant species (Tran et al., 2012; Jukanti, 2017). Such differences in the number of PPO genes are explained by the acquisition of new genes as a result of segmental duplications in plant genomes and by the loss of previously duplicated genes (Tran et al., 2012). Segmental duplications of *Ppo* genes are thought to have occurred in a common ancestor of monocotyledon species. Some of these ancestral genes have been lost during evolution; for example, in rice (*O. sativa*), only two *Ppo* genes have been identified (Yu et al., 2008). Alternatively, additional duplicated genes have been acquired; for instance, eight *Ppo* genes have been found in sorghum (Cai et al., 2013). Barley genes *Ppo1* and *Ppo2* are known to have arisen via segmental duplication of the long arm of chromosome 2 in a common ancestor of the *Triticeae* tribe (Taketa et al., 2010; Taranto et al., 2017). These genes—together with orthologous genes identified in maize, rice, sorghum, and *Brachypodium*—constitute the PPO-1 cluster (**Figure 2**). The other two barley genes, *Ppo3* and *Ppo4*, together with their orthologous genes form another cluster: PPO-2 (**Figure 2**). Because the PPO-2 cluster contains one *Ppo* gene each from maize and sorghum, and because separate orthologous genes for *Ppo3* and *Ppo4* have been identified only in common wheat (*T. aestivum*) and related species, we can hypothesize that these genes have arisen through a duplication that occurred in an ancestor of the *Triticeae* tribe.

Within the same plant species, *Ppo* family members are reportedly often differ in their spatial and temporal expression profiles among different vegetative and reproductive tissues and between baseline and a response to various environmental cues. Looking at the clusterization of *Ppo* genes in **Figure 2**, it is worth noting that barley genes *Ppo1* and *Ppo2* cluster together with the *S. bicolor Ppo-1* gene [*SbPpo3* according to the nomenclature of (Cai et al., 2013)], for which a thylakoid transfer domain was predicted, while barley genes *Ppo3* and *Ppo4* cluster with another sorghum gene: *Ppo-2* (*SbPpo8*), whose protein contains a predicted N-terminal signal peptide for targeting to the secretory pathway. From these data, one may conclude that the physiological functions of revealed *Ppo3* and *Ppo4* can differ from *Ppo1* and *Ppo2* belonging to different clades. Among the genes from the PPO-1 clade, wheat *Ppo1* and *Ppo2* genes have been reported to determine PPO activity in the grain and are considered targets for the breeding of PPO-deficient varieties (Beecher and Skinner, 2011). The rice *Phr1* gene (*PPO-1* of *O. sativa* in **Figure 2**), which belongs to clade PPO-1 too, participates together with *Bh4* in the control of black pigments’ synthesis in rice hulls (Fukuda et al., 2012) and determines phenol reaction phenotype (which could be revealed by immersing the grain in a phenol solution) (Yu et al., 2008). In barley, *Ppo1* and *Ppo2* control the phenol reaction phenotype of awns and grain, respectively (Taketa et al., 2010), while roles of these genes in melanin synthesis within the barley grain have not been studied. Because barley genes *Ppo1* and *Ppo2* cluster together with the *Phr1* gene, which controls the black hull trait in rice, one could say the functions of these genes are the same. Previously, enhanced total expression of both *Ppo* genes in grain envelopes combining hulls and pericarp tissues of the black-grained i:Bw*Blp1* line has been observed in comparison with white-grained Bowman (Glagoleva et al., 2017). Here, we distinguished and analyzed expression of each identified *Ppo* gene in combined tissues of hulls and pericarp (**Figure 3**) and in separated ones (**Figure 4**). It was found that *Ppo2* makes a major contribution to total *Ppo* expression in black grain envelopes; moreover, the expression of only this gene was higher in the presence of the *Blp1* locus. The enhanced transcriptional activity of the *Ppo2* gene in seed envelopes coincides with increased total PPO activity observed in whole mature grain of melanin-accumulating line (**Supplementary Figure 3**). The subsequent transcriptional assay of the *Ppo2* gene in detached hulls and pericarp tissues of hulled lines with melanin pigmentation and without it revealed enhanced transcription of *Ppo2* in black hulls and pericarp in comparison with the yellow ones. In naked sister lines, elevated transcription was observed in melanin-accumulating hulls, but not in pericarp in comparison with line without melanin. However, in both sets of lines (hulled and naked) transcription of *Ppo2* was significantly higher in hulls than in pericarp (**Figure 4**). Such differential expression of different members of one gene family in distinct tissues is a well-known phenomenon in plants. For example, among eight *S. bicolor Ppo* genes, only *SbPpo5* and *SbPpo7* were detected in the ovary or panicle tissues that developed under normal conditions, while the expression of the other genes (*SbPPO1*–*SbPPO3*, *SbPPO6*, and *SbPPO8*) was induced by stressful environments (involving a phytohormone or acid, alkaline, wounding, or heat shock treatments) (Cai et al., 2013). Among 11 *Ppo* genes of artichoke, three (*Ppo6*, *Ppo7*, and *Ppo11*) showed significant upregulation of transcripts in capitula tissues after cutting in comparison with the other genes; these genes were chosen as targets for CRISPR/Cas9-based inhibition of the enzymatic browning to maintain high quality of capitula after processing and during marketing (Pompili et al., 2023).

Although *Ppo2* demonstrated elevated transcription in both melanin-containing tissues—hulls and pericarp—in comparison with noncolored Bowman’s tissues, segregation analysis of two F_2_ populations derived by crossing the black-grained i:Bw*Blp1* line with barley specimens carrying mutations in coding part of *Ppo1* (variety 4970) or both *Ppo1* and *Ppo2* (Shogor 3) inferred the involvement of *Ppo2* in melanin formation in the hulls but not in the pericarp. In the first population (based on 4970), the segregation was not different from monogenic: 3:1, for which the *Blp1* locus is responsible (Woodward, 1941; Costa et al., 2001; Jia et al., 2017; Long et al., 2019). By contrast, in our second population (based on Shogor 3), the segregation ratio was 9:3:4, which corresponds to digenic mode of inheritance with the epistatic interaction between the two genes. Plants with the dominant *Blp1* gene and the recessive *Ppo2* gene were found to have an intermediate phenotype with light hulls and awns, which was designated by us as the gray phenotype. This observation allows us to propose the involvement of *Ppo2* in melanogenesis in hulls. In pericarp tissue that possesses dark pigmentation in the presence of recessive *Ppo1* as well as of *Ppo2*, it is possible that other oxidases participate in melanin formation under the control of the same *Blp1* gene. In this regard, multicopper oxidases called laccases should be considered too; they can oxidize a variety of phenolic substrates, and for these enzymes, functions beyond lignification have been proposed (Cai et al., 2006). In general, *Ppo2* is thought to be regulated by major gene *Blp1*, whose molecular functions have not been determined yet. Inactive PPO is not common among barley taxa. So far, only 51 phenol-negative barley accessions have been identified among 8894 samples analyzed, i.e., the grains do not discolor when immersed in a phenol solution (Takeda and Lin Chang, 1996). On the other hand, the loss of PPO activity and of dark pigmentation in seeds could have occurred during domestication of plants (Inoue et al., 2015; Balarynová et al., 2022). In rice, the allelic state of the *Phr1* gene is a taxonomic trait that separates two subspecies, *indica* and *japonica*, with recessive *Phr1* widespread within the *japonica* subspecies, and recessive *Bh4* within the *indica* subspecies (Yu et al., 2008). Crossing of uncolored specimens from *japonica* and *indica* leads to progenies with dark pigments of hulls (Fukuda et al., 2012). It is likely that in barley, inactive forms of PPO (in contrast to recessive *Blp1*) did not spread within a region where selection for white grains took place. The mutations in the *Ppo* genes may have occurred later in yellow-grained barleys and have not been under domestication-related selection in barley.

## Conclusions

Despite widespread occurrence of melanin pigments in nature, their synthesis in intact tissues of plants is not well understood. PPOs are considered the main participants of melanin production in damaged plant tissues when such an enzyme located in chloroplasts and its substrates located in vacuoles are released and interact giving rise to melanin. In undamaged tissues, such a mechanism of melanogenesis is not obvious, although there is evidence of the presence of substrates of PPOs in chloroplasts, and melanin granules in chloroplasts have been detected in some species. Here, the focus of the study is to test the hypothesis that *Ppo* genes are responsible for melanin formation in the barley grain, which accumulates melanin in the hulls and pericarp. The *Ppo* gene family was investigated, and two new genes coding for PPOs were found in barley and characterized for the first time. These genes proved to be functionally active and expressed in the barley spike. Among the four *Ppo* genes of barley, *Ppo2* was shown to be upregulated in melanin-accumulating hulls and pericarp in comparison with the tissues of the line, not accumulating melanin, but transcription in hulls was significantly higher than in the pericarp. Moreover, recessive *Ppo2* in combination with dominant *Blp1* resulted in hybrids with discolored hulls and a dark pericarp. Although our results imply the involvement of *Ppo2* in melanization of barley hulls, definitive proof can be obtained only by knocking out *Ppo2* in black-grained barely genotypes by genetic engineering techniques.

## Data availability statement

The original contributions presented in the study are included in the article/**Supplementary Materials**, further inquiries can be directed to the corresponding author/s.

## Author contributions

AG: Formal Analysis, Investigation, Methodology, Validation, Writing – original draft. TK: Investigation, Methodology, Resources, Writing – review & editing. EK: Conceptualization, Writing – review & editing. OS: Conceptualization, Funding acquisition, Writing – review & editing.

## Supplementary material

Supplementary Table 1 The list of primers used in the study.

Supplementary Table 2 Identification numbers of *Ppo* genes from the Ensembl Plants and NCBI databases used for the phylogenetic analysis.

Supplementary Figure 1 The scheme of obtaining of NILs i:Bw*nud1* and i:BwBlp1*nud1*. Lines with a dark-blue aleurone and i:Bw*Blp1* lines based on the cv. Bowman genetic background were used. PCR primers specific to genes *Blp1* and *Nud1* were employed for marker-assisted selection.

Supplementary Figure 2 The alignment of amino acid sequences of PPO.

Supplementary Figure 3 PPO activity assay in cv. Bowman and *i:BwBlp1* line. A405 means absorbance at 405 nm wavelength after incubation of grinded seeds in L-tyrosine solution. * - significant differences between samples (*U* test, *p* < 0.05).

Supplementary Figure 4 Electropherograms of the *Ppo2* gene alleles screening in F_2_ population of Shogor 3 × i:Bw*Blp1* cross. The amplicons lengths: 88 bp for dominant allele of the *Ppo2* gene, 96 bp for the recessive one.
