# Supplementary figures and images for: Polyphenol oxidase genes in barley (Hordeum vulgare L.): functional activity with respect to black grain pigmentation
Source: Front Plant Sci. 2024 Jan 8;14:1320770. doi: 10.3389/fpls.2023.1320770 (PMC10800887; doi:10.3389/fpls.2023.1320770)

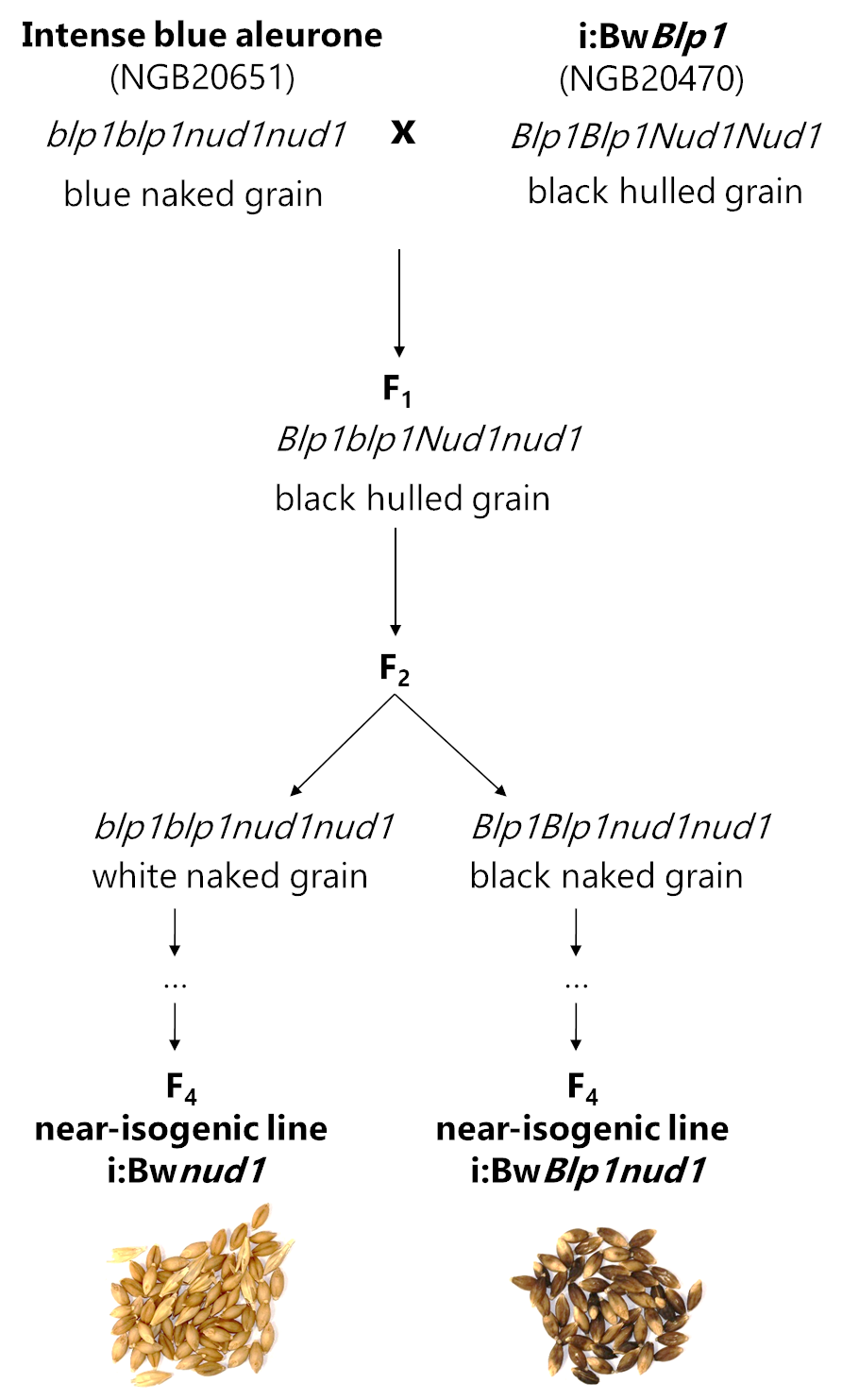

Supplement: Supplementary Figure 1 — The scheme of obtaining of NILs i:Bwnud1 and i:BwBlp1nud1. Lines with a dark-blue aleurone and i:BwBlp1 lines based on the cv. Bowman genetic background were used. PCR primers specific to genes Blp1 and Nud1 were employed for marker-assisted selection. [file Image_1.tif]

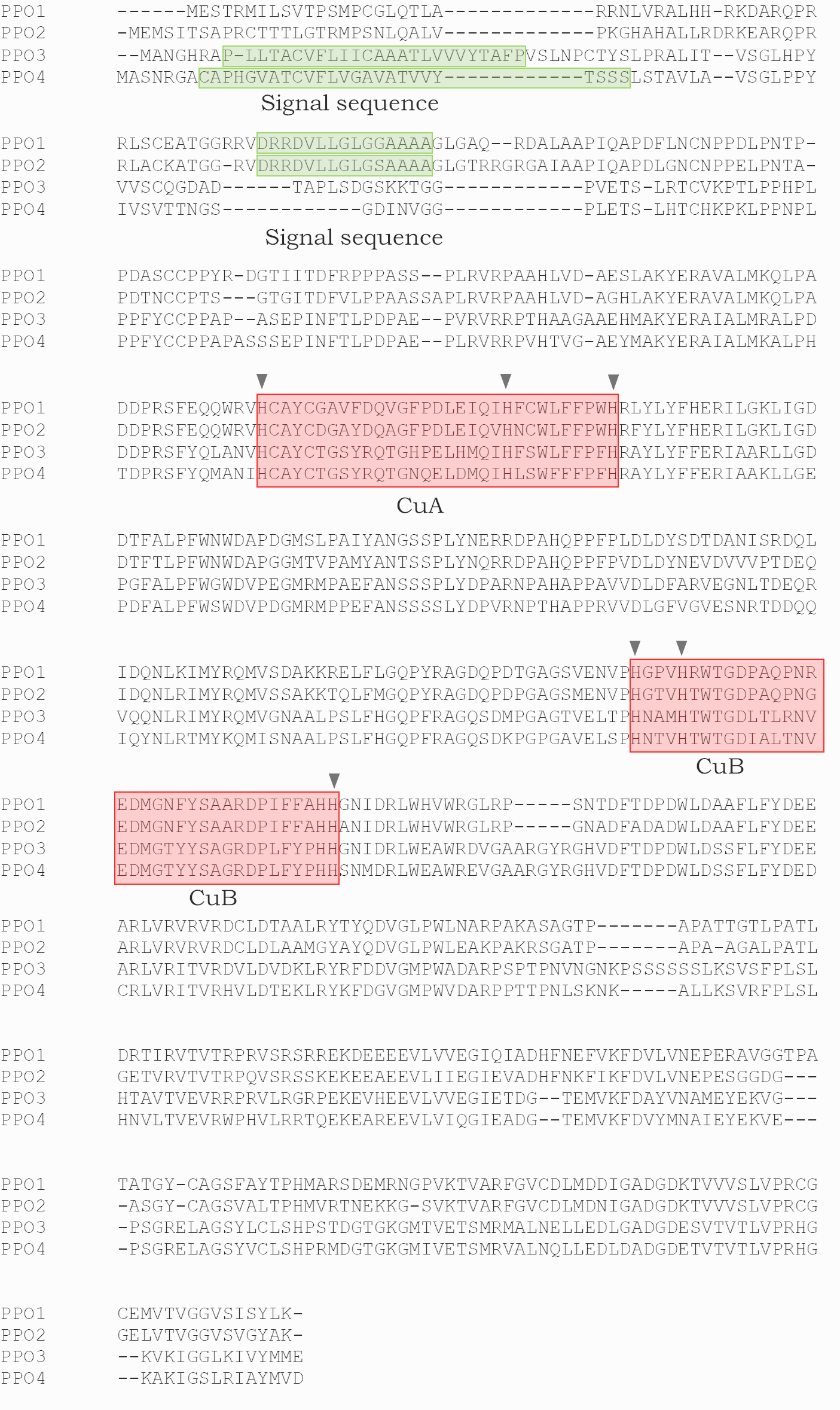

Supplement: Supplementary Figure 2 — The alignment of amino acid sequences of PPO. [file Image_2.tif]

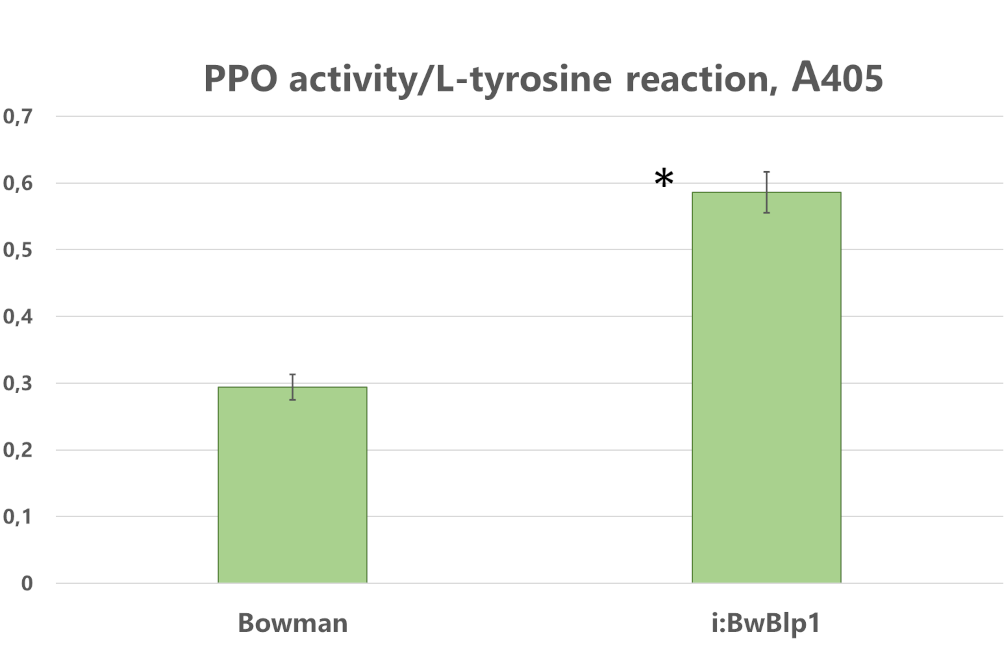

Supplement: Supplementary Figure 3 — PPO activity assay in cv. Bowman and i:BwBlp1 line. A405 means absorbance at 405 nm wavelength after incubation of grinded seeds in L-tyrosine solution. * - significant differences between samples (U test, p < 0.05). [file Image_3.tif]

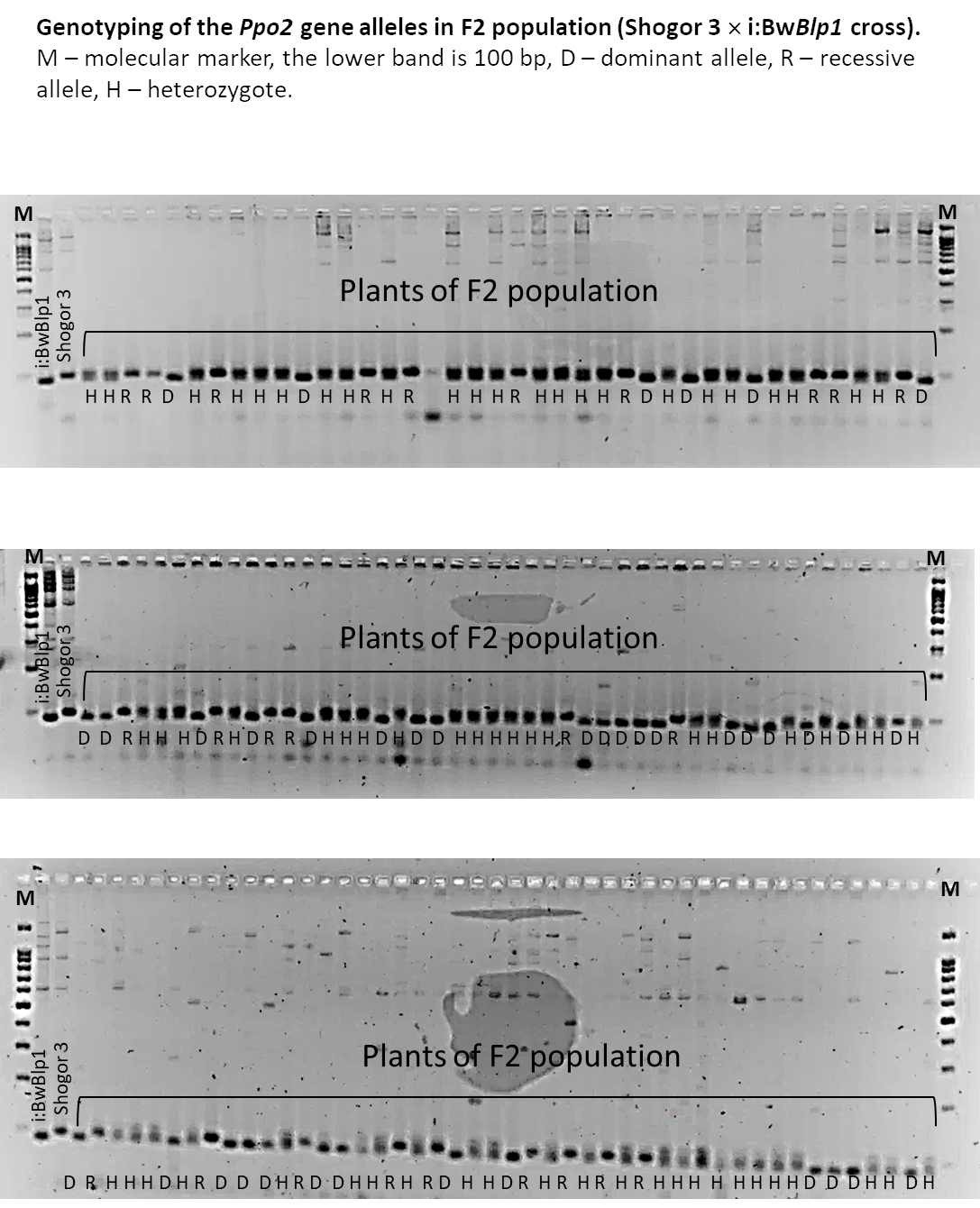

Supplement: Supplementary Figure 4 — Electropherograms of the Ppo2 gene alleles screening in F2 population of Shogor 3 × i:BwBlp1 cross. The amplicons lengths: 88 bp for dominant allele of the Ppo2 gene, 96 bp for the recessive one. [file Image_4.tif]
